# Contribution of Interleukin-17A to Retinal Degenerative Diseases

**DOI:** 10.3389/fimmu.2022.847937

**Published:** 2022-03-22

**Authors:** Huimin Zhong, Xiaodong Sun

**Affiliations:** ^1^ Shanghai General Hospital, Shanghai Jiao Tong University School of Medicine, Shanghai, China; ^2^ National Clinical Research Center for Eye Diseases, Shanghai, China; ^3^ Shanghai Key Laboratory of Ocular Fundus Diseases, Shanghai, China; ^4^ Shanghai Engineering Center for Visual Science and Photomedicine, Shanghai, China; ^5^ Shanghai Engineering Center for Precise Diagnosis and Treatment of Eye Diseases, Shanghai, China

**Keywords:** retinal degenerative diseases, interleukin-17A, age-related macular degeneration, diabetic retinopathy, glaucoma

## Abstract

Retinal degenerative diseases are a leading cause of vision loss and blindness throughout the world, characterized by chronic and progressive loss of neurons and/or myelin. One of the common features of retinal degenerative diseases and central neurodegenerative diseases is chronic neuroinflammation. Interleukin-17A (IL-17A) is the cytokine most closely related to disease in its family. Accumulating evidence suggests that IL-17A plays a key role in human retinal degenerative diseases, including age-related macular degeneration, diabetic retinopathy and glaucoma. This review aims to provide an overview of the role of IL-17A participating in the pathogenesis of retinal degenerative diseases, which may open new avenues for potential therapeutic interventions.

## Introduction

Degenerative retinal diseases affect millions of people worldwide and can eventually lead to irreversible vision loss. Currently, the prevalence of degenerative retinal diseases such as AMD, DR or glaucoma is increasing year by year (1). It is estimated that around 288 million people worldwide will be affected by AMD by 2040 (2). The raw prevalence of blindness due to DR increased substantially between 1990 and 2015 due to the rising prevalence of type 2 diabetes (3). Additionally, more than 76 million people are estimated to have glaucoma globally (4). There is no effective treatment that can reverse these degenerative processes. Retinal neurons continue to withstand long-term light, stress and aging or other factors leading to degenerative changes. Even though healthy retinas can resist stress for decades, the risk of retinal dysfunction affected by the disease gradually increases (5). A large body of convincing evidence demonstrates that inflammatory factors have been qualified as an important role in retinal degeneration (6–9).

IL-17A is the first member of the IL-17 family to be identified because it is most closely related to human health and disease (10). IL-17A was screened out from subtracted cDNA library in 1993, and its binding receptor was first discovered in 1995 (11, 12). At present, IL-17A has been observed under different tissues and immunopathological conditions, such as autoimmune diseases, tumors or obesity (13). The expression level of IL-17A cytokines is closely related to the severity and progression of the disease in human neurodegenerative diseases (9, 14, 15). Clinically, it was found that the levels of IL-17A in the plasma and cerebrospinal fluid of Alzheimer’s disease patients were elevated (16, 17). In addition, dopaminergic neurodegenerative diseases, dyskinesias, and blood-brain barrier destruction in *Il-17a* ^-/-^ mice were all alleviated (18). Interestingly, Yang et al. (19) reported that IL-17A did not cause neuroinflammation aggression. Overexpression of IL-17A could also reduce the level of soluble Aβ in the mid-cerebrospinal fluid and hippocampus as well as improving glucose metabolism, which provided a more comprehensive basis for the pathogenesis of multiple sclerosis and experimental autoimmune encephalomyelitis (19).

On the other hand, there are more and more researches on IL-17A in eye diseases. The activation of IL-17 pathway has become an important target of autoimmune uveitis, pathological neovascularization and other diseases (20–22). In fact, the mechanism of IL-17A is more complicated than simply causing inflammation. Its contribution to the specific conditions of health and disease has only just begun to be recognized. The role of IL-17A in retinal degenerative diseases is still unclear. Therefore, this article mainly reviews IL-17A and its role in retinal degenerative diseases.

## IL-17 Family

In 1993, Interleukin (IL) -17A (formerly CTLA8) was described and named by Rouvier et al. for the first time (11, 23). Subsequently, IL-17 family has grown gradually, with six cytokine members from IL-17A to IL-17F. (24) In addition, there are five receptor subunits in the family, including IL-17RA to IL-17RE (25). Little is known about IL-17B to IL-17F, while IL-17A has been widely studied because it has the closest relationship with human health and disease. IL-17A is the primary cytokine in the IL-17 series, originally discovered to be produced by activated CD4 ^+^ memory T lymphocytes. Subsequently, it was later discovered that it can also be the product of CD8 ^+^ memory T lymphocytes (23, 26–28). Although most of IL-17A is produced by activated T lymphocytes, other inflammatory cells such as neutrophils, macrophages and even microglia may also be producers of IL-17A under certain circumstances (29–31). IL-17A is a key cytokine responsible for the recruitment, activation and migration of neutrophils, which can be combined with IL-17RC/IL-17RA on fibroblasts, endothelial cells and epithelial cells in the form of dimers to induce pro-inflammatory secretion of the medium (32, 33).

IL-17A is often considered as a bad character. Alzheimer’s disease (AD) is a neurodegenerative disease dominated by amyloid deposition in the brain. Recent studies have reported that IL-17A was involved in the early pathological process of AD, causing cognitive impairments and synaptic dysfunction (34) Neutralization of IL-17A restored the function of Aβ-induced neuroinflammation and memory impairment (35). Animal experiments have also confirmed that IL-17A functioned in the occurrence and development of Parkinson’s disease (PD). Dopaminergic neurodegeneration, dyskinesia, and BBB disruption were ameliorated in *Il-17a*-deficient mice (18). In addition, increased levels of IL-17A were detected in samples from both MS and ALS patients (15, 36). Likewise, IL-17A is playing an increasingly important role in inflammatory autoimmune and cardiovascular disease (37–39). Interestingly, on the other hand, Reed et al. demonstrated that IL-17A could improve social deficits in a mouse model of neurodevelopmental disorders (40). IL-17A even promoted wound healing, which not only providing antibacterial protection and pathogens elimination, but also encouraging the proliferation of corneal forming cells after injury (13, 41, 42). Therefore, the above findings suggest that IL-17A is not just an inflammatory factor. IL-17A may protect the body under certain circumstances, but in those with chronic or degenerative conditions, IL-17A tends to start causing disease.

IL-17A is mediated by IL-17F, which normally co-produces IL-17A with T helper 17 (Th17) cells. The percentage homology between IL-17A and IL-17F is 50%, and they are co-expressed on linked genes (10, 43). Nevertheless, IL-17A and IL-17F have similar biological activities but different functions. IL-17A is involved in autoimmunity, inflammation and tumorigenesis while IL-17F is mainly involved in the mucosal defense mechanism. What’s more, IL-17A has a stronger affinity for IL-17RA/RC complex so it can promote the induction of pro-inflammatory genes more than IL-17F. Studies showed *IL-17F* mRNA instead of *IL-17A* mRNA could be expressed on colonic epithelial cells (44). IL-17F has been found to be an effective target for the treatment of colitis because *Il-17f^–/–^* mice may resist the development of colitis (45). Studies have shown that IL-17F is more highly expressed in psoriasis and spondyloarthritis than IL-17A (46). It was demonstrated that IL-17F had similar pathogenic effects as IL-17A in β-cell lines and pancreatic islets in type I diabetic mouse models (47). In addition, IL-17F may also be involved in systemic sclerosis fibrosis and vascular disease (48). Dual inhibition of IL-17A and IL-17F may be more effective than IL-17A alone in reducing disease pathological changes (49, 50).

Screening for IL-17A to identify homologous genes led to the discovery of IL-17B whose mRNA is strongly expressed in the adult digestive system (51). Zhou et al. (52). found that the concentration of IL-17B in adults and children with community-acquired pneumonia was significantly increased. In addition, the level of IL-17B in patients with lupus erythematosus was significantly higher than that in the control group, involving in the pathogenesis of the disease. At the same time, more and more evidences have proved that IL-17B/IL-17RB get trapped in the occurrence and poor prognosis of patients with malignant tumors such as pancreatic cancer, gastric cancer, lung cancer and breast cancer (53).

The uniqueness of IL-17C lies in its cell source: epithelial cells rather than CD4^+^ cells are the producer of IL-17C, whose receptor IL-17RE is expressed in Th17 cells and the epithelial cells themselves. New research showed that IL-17C maintained the autocrine cycle in the epithelium, thus inhibiting innate immune infection or binding to receptors to activate the adaptive immune response (54). The usage of anti-IL-17C antibodies could control skin inflammation in mouse models of psoriasis and atopic dermatitis (55, 56). In addition, inhibiting IL-17C or blocking IL -17 RE might be a new treatment for acute kidney injury (57). Other studies have shown that IL-17C mediated antibacterial effects on pseudomonas aeruginosa by interfering with iron absorption in the nasal epithelium (58).

IL-17D possessed the similarity of inducing increased expression of chemokines and cytokines with other members in family (59). Curiously, IL-17D has not been found in the synovial fluid or peripheral blood of patients with rheumatoid arthritis but detected in its nodules. In addition, *IL-17D* mRNA expression in the skin of psoriatic patients was surprisingly reduced. IL-17D induces anti-tumor effects by recruiting NK cells (60–62).

More and more evidences showed that IL-17E (IL-25)was a “barrier cytokine” whose expression depends on external environmental factors. It may cause inflammatory diseases, such as atopic dermatitis, inflammatory bowel illness or asthma when upregulating. IL-17E has been proved to stimulate the proliferation and metabolic activity of keratinocytes. Excessive secretion of IL-17E caused by irritant atopic dermatitis (63, 64).

**Figure 1:**
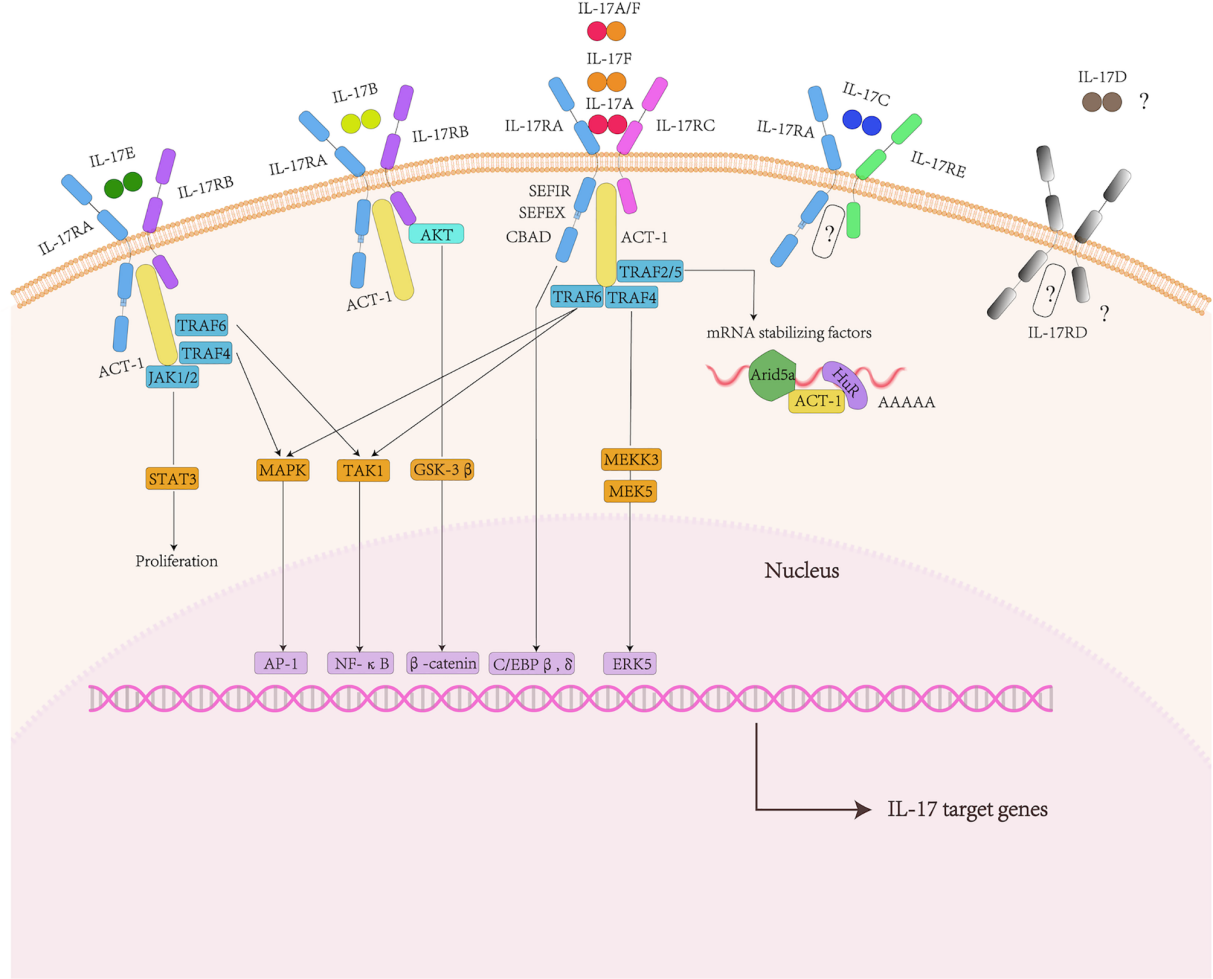
IL-17 family and signaling pathway. The IL-17 family includes six factors and five receptors. IL-17RA and IL-17RC subunits bind to IL-17A, IL-17F and IL-17AF ligands. IL-17RA and IL-17RB subunits bind to IL-17B and IL-17E ligands. The receptors of IL-17C are IL-17RA and IL-17RE. IL-17D and IL-17RD are currently unknown. They shared the common cytoplasmic motif SEFIR domain can recruit act1 and nonconserved region called SEFEX.ACT-1 can bind to TRAF family proteins. For IL-17A, TRAF6 can activate NF-κB pathway, MAPK/ap1 pathway. Act1 can also promote post-transcriptional mRNA stabilization through the transcriptional induction of TRAF2/5. In addition, The Il-17R-act1 complex binded to MEKK3 and MEK5 *via* TRAF4 to activate ERK5. In addition to the classic pathway, IL-17E also mediates Act1-JAK1/2-STAT3 pathway.

In a word, members of the IL-17 family play a key role in inflammatory diseases, autoimmune diseases and cancer. It regards IL-17 family members and their receptors as potential targets for future drug treatments (**Figure 1**).

## IL-17 Family-Related Signaling Pathway

The IL-17R family contains five receptor subunits:IL-17RA to IL-17RE. All receptor subunits are type I transmembrane proteins. IL-17RA is a founding member of the IL-17R family as well as the co-receptor used by several other IL-17 family ligands. They shared the common cytoplasmic motif called the similar expression of fibroblast growth factor and IL-17R (SEFIR) domain.

Besides, there existed nonconserved region called SEFIR-Extension” (SEFEX) (65). Unlike other IL-17 family receptors, IL-17RA contained another C/EBP β activation domain (CBAD), whose function was related to negative signal regulation (66). Therefore, IL-17RA had at least two structurally and functionally discontinuous signal regions in the cytoplasm to control downstream signal events.

The IL-17B signaling pathway is mainly reflected in cancer diseases. IL-17B in breast cancer cells activated ERK and NF-kB pathways and enhanced the expression of anti-apoptotic Bcl-2 family members (67, 68). AKT/GSK-3β/β-catenin signaling pathway was promoted by IL-17B/IL-17RB signaling pathway to up-regulate Sox2, Oct4 and Nanog proteins, inducing stem cell transformation and epithelial to mesenchymal transformation of gastric cancer and lung cancer cells (69, 70). There are still unknown about receptor of IL-17D and the ligand of IL-17RD. Currently, the signaling pathways regarding downstream of IL-17C are rarely reported (53).

Among the IL-17 family cytokines, the signaling pathways concerning about IL-17A and IL-17E are the most fully characterized. IL-17A transmits signals through the IL-17RA and IL-17RC receptor subunits. IL-17F and IL-17A/F heterodimers also bind to this receptor complex.

The initial binding of IL-17A to its receptor complex recruited the adaptor protein ACT1, which interacts through the shared SEFIR domain. The next step was ACT1 to identify and ubiquitinate different TNF-receptor–associated factor (TRAF) adaptors (71). The activation of TEAF6 drived the triggering of the classical TAK1/NF-κB pathway, c/EBPβor c/EBPδ transcription factors and MAPK/AP-1 pathway (23, 72–75). The IL-17R-ACT1 complex binded to MEKK3 and MEK5 *via* TRAF 4 to activate ERK5. IL-17 signaling promoted the regulation of post-transcriptional IL-17 target gene mRNA stability or controlled a variety of RNA binding proteins through ACT1- TRAF2- TRAF5 complex (including HuR and Arid5a) (13).

IL-17RB, the binding receptor of IL-17E, needs to combine with IL-17RA to form a complex to mediate the downstream signal cascade in target cells (76). The supplementation of TRAF6 was essential for IL-17E-mediated activation of the NF-kB pathway (72). The activation of MAPKs may depend on TRAF4 (77). In addition, IL-17E has been also shown to activate the Act1-JAK1/2-STAT3 pathway, leading to the proliferation of keratinocytes and the production of inflammatory cytokines and chemokines in mouse skin (63).

Many regulators amplify or inhibit inflammation mediated by IL-17, respectively. Understanding its mechanism of action may provide strategies for designing new interventions to treat IL-17-mediated signals and inflammation (**Figure 1**).

## Inflammation and Retinal Degenerative Diseases

The retina is a tissue with immune properties, whose blood-retinal barrier and immunosuppressive microenvironment can maintain its homeostasis. In addition to physical barrier defenses, the retina is also protected and monitored by autoimmunity, namely microglia and the complement system (78). Ageing increases the risk of various retinal degenerative diseases, such as age-related macular degeneration, diabetic retinopathy, and glaucoma. All age-related retinal diseases have two common features:destruction of retinal homeostasis and low-grade chronic inflammation (79–81).

Resident microglia can be regarded as the immune guards of the retina. Inflammatory factors recruited by microglia accumulated in the damaged layer play a crucial role in the pathological changes (79, 80, 82). Drusen, the pathological marker of AMD, attracted macrophages and microglia (83, 84). The patients with geographic atrophy also showed microglia that positively swallowed photoreceptor fragments (85). It displayed that activation of microglia and macrophage were early and long-term chronic features in the pathogenesis of AMD (86). In human glaucoma, the activated microglia morphology was found in the optic nerve head and the choroidal retinal area adjacent to the optic nerve head (87). In addition, microglia expressed TNF-α and several metalloproteinases including MMP-1, MMP-2, MMP-3 and MMP-14, which were significantly increased during optic neuropathy (88). Therefore, activation of microglia may disrupt tissue stability during the early changes in the glaucoma disease process. Zeng et al. (89)deeply studied the different stages of 21 diabetic retinopathy patients’ eyes, and found that the neovascularization was very severely surrounded by microglia. Reactive microglia participated in all stages of diabetic retinopathy, even promoting its evolution to a proliferative state (89).

**Figure 2:**
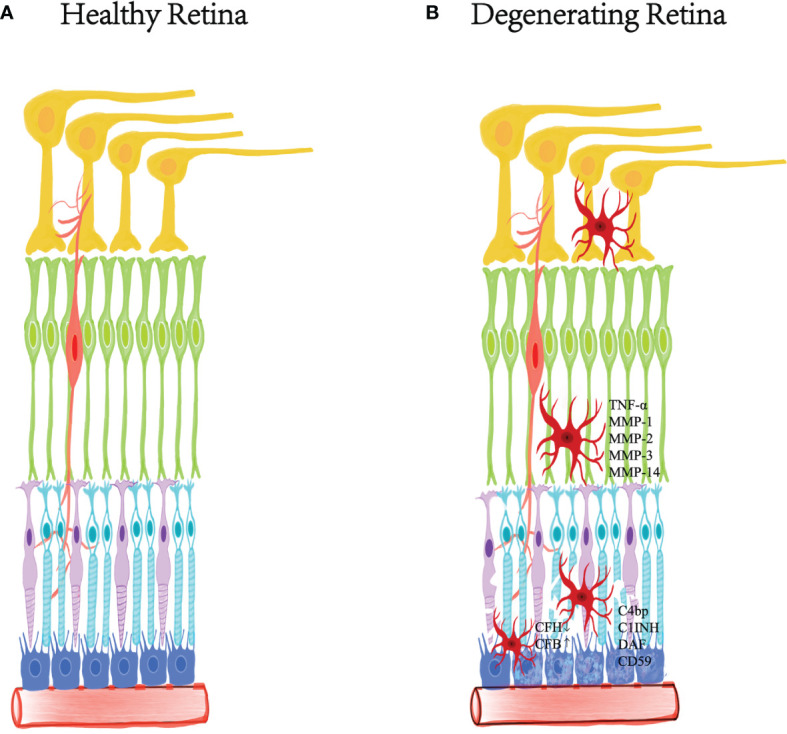
The relationship between inflammation and degenerative retina. Degenerative retinal diseases are characterized by the destruction of retinal homeostasis and mild chronic inflammation. Inflammatory factors released by microglia recruited in the injured layer play a crucial role in pathological changes, expressing TNF-α and several metalloproteinases, including MMP-1, MMP-2, MMP-3 and MMP- 14. Microglia, retinal pigment epithelial cells and neurons in the retina express various complements and complement regulators. The degenerative retina reduces the expression of complement inhibitor CFH, while enhancing the expression of CFB in RPE cells. In addition, changes in the immunosuppressive microenvironment can interfere with RPE and microglia to produce various complement regulators (C4bp, C1INH, DAF and CD59). **(A)** The healthy retinal layered morphology. **(B)** The damaged retina under degenerative disease.

The retina has a complement regulation system. Actually, microglia, retinal pigment epithelial cells (RPE) and neurons in the retina express various complement and complement regulators (78, 90, 91). Under physiological conditions, retinal cells always expressed relatively high levels of complement regulators (CFH, C1INH, and CD59) and low levels of complement proteins (such as C1q, c3, and CFB) (92–95). Oxidative stress and inflammation attenuated the expression of complement inhibitors but increased the expression of complement components during aging (79, 92, 94–96). It means that the complement system may function in the immune defense of the retina. Age-related oxidative damage could directly regulate the expression of retinal cell complement. For example, oxidized photoreceptor outer segments not only reduced the expression of complement inhibitor CFH, but also strengthened the expression of CFB in RPE cells (97, 98). On the other hand, the change of the immunosuppressive microenvironment was able to interfere with the production of various complement regulators (C4bp, C1INH, DAF, and CD59) by RPE and microglia. In addition, the inflammatory factors released by subretinal macrophages may up-regulate the expression of complement protein in RPE cells (92, 99) (**Figure 2**).

## IL-17A and Retinal Degenerative Diseases

### Age-Related Macular Degeneration (AMD)

Age-related macular degeneration (AMD) is the main cause of irreversible blindness in the elderly, affecting 8.7% of people worldwide. AMD refers to the age-related progressive degeneration of photoreceptors and underlying RPE in the macular area of the retina (2, 100). Oxidative stress, inflammation and heredity are all considered to be the critical pathogenic factors leading to the occurrence of AMD (101–103). Among them, more evidences prove the important role of immune inflammation in the progression and treatment of AMD.

The IL-17 signaling pathway may be involved in one of the important pathogenesis of AMD (104). The study found that the IL-17A level of AMD patients’ serum, especially neovascularization and geographic atrophy AMD patients, was significantly higher than that of the normal group (105, 106). It has been reported that there were 456 differentially expressed genes (DEGs) and 4827 differentially methylated CpGs (DMCs) in the RPE samples of 26 AMD patients and 105 normal people in the GEO and Array Express databases. Enrichment analysis showed that up-regulated genes got involved in IL-17 signaling pathway in AMD (107). In terms of experiments, RPE was induced by fungi to induce different natural immune responses. After infection of ARPE-19 cells with Aspergillus flavus, *IL-17A* was increased by 5.6 times (108). Liu et al. (109) have proved that complement C5a in the serum of AMD patients promoted the production of Th17 family cytokines. However, there is no evidence that the growth of Th17 cytokine levels in AMD patients’ serum is directly caused by the increased expression of C5a. Other unknown factors that may also cause the activation of T cell in AMD patients (109). The activation of inflammasomes is one of the key causes of AMD. The mechanism by which IL-17A activated NLRP3 and secreted IL-1β is related to the production of reactive oxygen species. IL-17A induces Akt, ERK1/2, p38 MAPK and NF-κB p65 phosphorylation signaling pathways in RPE cells. Blocking NF-κB had an effect on IL-17A-induced IL-1β mRNA expression. It has been confirmed that NLRP3 was the reactive inflammasomes responding to IL-17A and IL-17A promotes the expression of caspase-1 and NLRP3 mRNA from the post-transcriptional level (110). Other studies also showed that the IL-17 signaling pathway got involved in the oxidative stress of human RPE cells through the targeted pathway of 4-octylitaconate (111). IL-17A has been proved to induce RPE cells death by activating the pro-apoptotic caspase-3 and caspase-9 pathways due to the accumulation of cytoplasmic lipids and autophagosomes *in vitro* experiments (105). The blood-retinal barrier is composed of RPE cells, which is one of the blocking factors that prevent abnormal components from choroid from entering the retina. ARPE-19 cells were stimulated by IL-17A to destroy the barrier function between RPE cells through activating the JAK1 signaling pathway so that the distribution of tight junction zone occlusion protein-1 and occludin disordered (112, 113). The protein expressed by RPE may take participate in the angiogenesis of choroidal endothelial cells (CECs). IL-17A exerted its ability to mediate the expression of CCL2 and CXCL8 by stimulating RPE *in vitro* so as to promote the proliferation and migration of CECs and the formation of capillary-like structures (114).

Interestingly, IL-17A with diametrically opposite performance depending on the cells stimulated. The responding IL-17A of retinal astrocytes cells generated more pro-inflammatory cytokines and chemokines, leading to increased migration of granulocytes. Whereas, IL-17A at the same concentration would express more suppressor of cytokine signaling proteins, thereby attenuating the production of pro-inflammatory cytokines and chemokines (115). The duality of IL-17A remains to be verified, but it could provide clues for new therapeutic targets and better prognosis.

On the other hand, ARPE-19 cells themselves constitutively express IL-17RC instead of IL-17RA (112). However, it was also found that *IL-17RA*, the main receptor of IL-17 signaling, was one of the most up-regulated inflammatory genes in human RPE cells after exposure to oxidative stress. In words, human primary RPE cells expressed both IL-17RC and IL-17RA. A significant increase in *Il17ra* was also detected in the retina of AMD-like mouse models so that knockout of *IL-17RA* in RPE cells can inhibit cell apoptosis and reduce inflammation. Transcription factor KLF4 promoted the production of other inflammatory factors by directly activating IL17RA expression in RPE cells (110, 116).

In addition, IL-17RC methylation could act as a marker for the degeneration of RPE cells and choroidal neovascularization (CNV) *in vitro*. There is a significant correlation between promoter methylation status and expression of IL-17RC. The mRNA overexpression of IL-17RC in RPE cells was 6.3 times higher than that under normoxia conditions due to the demethylation of the IL-17RC promoter in chemical hypoxia. At the same time, the epigenetic control of IL-17RC by hypoxia-inducible factor-α and hypoxia could synergistically enhance the activation of angiogenic factors (117). Using siRNA to knock out *Il-17rc* may inhibit the apoptosis of RPE activated by IL-17A. Gene therapy with adeno-associated virus vectors encoding soluble IL-17A receptors prevented IL-17-dependent retinal degeneration and had certain therapeutic potential for AMD (105).

There is a strong link between inflammation and the development of AMD. Both IL-17 and its receptor are considered biomarkers of the disease in AMD patients. IL-17A-targeted therapy is being explored as the most likely therapeutic strategy in the clinic.

### Diabetic Retinopathy (DR)

Diabetic retinopathy (DR) is a complication of diabetes in the eye disease, and also one of the main causes leading to blindness in the world. The study of DR is of great importance because of the prevalence of diabetes. IL-17A and Th17 cells have been confirmed to participate in various types of diabetes and accumulate multiple organ complications (39, 118–121).

Nadeem et al. (122) found that the concentration of IL-17A in the serum of DR patients was increased compared with that of healthy people. In addition, another study clarified that the level of IL-17A in the vitreous fluid of DR patients was significantly higher than that of normal one so that disturbances in Th17 cells and IL-17A levels may be related to DR (123). The same results were found when comparing serum and vitreous samples from patients with proliferative diabetic retinopathy (PDR) (124). Clinically, proprietary Chinese medicines are usually used to treat PDR. Both compound xueshuantong capsule and hexuemingmu tablet contain ingredients that served as the treatment of PDR, and mainly affected the following pathways: response to oxidative stress, vascular regulation and blood coagulation. Network pharmacology analyzed that IL-17 signaling pathway ranked in the top five pathways with the most significant enrichment and the highest gene ratio (125).

IL-17A induced endothelial cells to secrete inflammatory factors, followed by down-regulation of tight junction proteins to promote retinal inflammation and blood-retinal barrier (BRB) destruction (112). BRB dysfunction underlies diabetic macular oedema under sight-threatening conditions. Inflammation plays an important role in BRB dysfunction. IL-17A damaged BRB by activating the JAK1 signaling pathway. Targeting this pathway may be a new method to treat inflammation-induced diabetic macular oedema (113). The more widely used DR-like pathology model is induced by high glucose (HG), ARPE-19 cells were induced by HG to simulate the breakdown of BRB, leading to the higher levels of IL-17A. The addition of insulin-like growth factor-2 inhibitors could improve the inflammatory invasion of IL-17A into cells to achieve a protective effect (126). In addition, HG induces the expression and secretion of IL-17A and IL-17RA in primary Müller cells. IL-17A further enhanced the activation of Act1/TRAF6/IKK/NF-κB signaling pathway in HG-treated Müller cells and Ins2Akita diabetic mouse. It has been established that Müller cells enhance inflammation and neuronal apoptosis in the retina through an autocrine signaling cascade (127, 128).

IL-17 acts as a powerful proinflammatory cytokine and serves for mobilizing neutrophils, then inducing the secretion of inflammatory factors (129, 130). In addition, the interaction of these mediators attenuates the tight junction protein cake leading to the disruption of BRB (112). Diabetes-induced retinal vascular leakage may be mediated by IL-17A regulation of neutrophil elastase and its activity because *Il-17a* gene deletion greatly attenuated the increase in neutrophil elastase levels of diabetic-retina (131). IL-23 could promote IL-17A secretion by Th17 cells so that the current study of the IL-23/IL-17 axis is increasingly being studied in DR (132–134). Streptozotocin (STZ) -induced rat model demonstrated that IL-17A levels in both retina and serum were greatly increased. After intravitreal intervention using IL-23Rp19 antibody, the BRB of RPE cells was restored and IL-17A was down-regulated (133). Namely, the significance was that blocking the IL-23/IL-17 axis could delay the process (135). On the basis of Th17 cells causing retinal inflammation, vascular leakage and capillary degeneration in the retina of diabetic mice, Zapadka et al. further demonstrated that aromatic hydrocarbon receptor agonists (VAF347) can alleviate the pathophysiological process by inhibiting Th17 cell differentiation and the production of IL- 17A. (120, 136, 137)

IL-17A has been shown to play a role in promoting angiogenesis in ischemic retinopathy. The interaction between IL-17A and endoplasmic reticulum stress promoted RNV by regulating the TXNIP/NLRP3 signaling pathway in macrophages under hypoxic conditions. *In vitro*, macrophages isolated from the retina of a mouse model of oxygen-induced retinopathy have also been verified to activate the NLRP3 inflammasome through the IL-23/IL-17 axis to promote the formation of RNV (138).

IL-17A-expressing T cells and neutrophils were adhered to the retinal vasculature. IL-17A could bind to its receptors (IL-17RA) expressed on photoreceptors, Muller glia, and retinal endothelial cells, then initiating a downstream IL-17A-dependent injury mechanism (120, 127, 128, 136). Various pathological features mediated by diabetes were significantly reduced in *Il-17a ^−/−^* mice (136). Cells with RORγt expression may be qualified as the main makers of IL-17A in the retina. IL-17A production prevented by the RORγt inhibitor SR1001 in mice, thus successfully blocking STZ-induced retinal inflammation and retinal endothelial cell death (120, 139, 140). In addition, Lindstrom et al. (120) studied IL-17A-dependent apoptotic signaling cascades in the RPE. After initiating receptor signaling, IL-17A induce constitutive expression of Act1 on the RPE and recruit FADD to interact with it, further directing the activation of apoptotic proteins caspase 8 and caspase 3. IL-17A got involved in capillary degeneration through the apoptotic signaling pathway in endothelial cells (120).

IL-17A is a hallmark cytokine produced by Th17 cells and may achieve neovascularization through a regulatory network of cytoskeletal remodeling, vascular endothelial growth factor (VEGF), VEGF-related cytokines, and complement components (141). It has been found that IL-17A may induce Müller cells to produce elevated levels of VEGF during early DR (120). DR-mediated IL-17A production was dependent on RORγt. The RORγt small molecule inhibitor (SR1001) served as a candidate for DR therapeutics for SR1001 had properties that could cross the BRB (139). In addition, IL-17A has been implicated in resistance to anti-VEGF agents. Anti-VEGF agents were currently exerted to treat different types of cancer, wet AMD, macular edema, and DR (142–146). The usage of IL-17A neutralizing antibodies significantly improved the antitumor activity of anti-VEGF therapies in cancer research. (147). Similarly, diabetes-mediated IL-17A enhanced VEGF production so that preventing IL-17A production may be a potential treatment to delay the progression of DR. Collectively, elevated IL-17A levels can lead to local inflammation and immune responses in the retina, which may promote the development of DR.

### Glaucoma

Glaucoma is a neurodegenerative disease closely related to age. It is characterized by increased intraocular pressure due to obstruction of the outflow of aqueous humor, which causes progressive damage to the optic nerve (148). Glaucoma is usually defined as a disease of the optic nerve, which is formed by the axons of retinal ganglion cells (RGCs). The death and loss of RGCs is the greatest threat to this disease, and finally leading to irreversible blindness (149, 150). The treatment is usually through the use of topical drugs or surgical intervention to reduce intraocular pressure. Drug therapy includes two aspects. One is to increase the drainage of ocular fluid, such as the use of prostaglandin analogs, Rho kinase inhibitors, nitric oxides or miotics. The other is to reduce the amount of ocular fluid produced, such as the commonly used clinical drugs include α-adrenergic agonists, beta blockers, and carbonic anhydrase inhibitors, etc. (151, 152). However, the control of intraocular pressure cannot completely avoid the death of RGC (153). Therefore, other mechanisms such as neuroinflammation may also be related to the progression of glaucoma damage (154, 155). The role of immune-inflammatory response in glaucomatous optic nerve damage has received increasing attention and has become a research hotspot (156–159). Reports have showed that the inflammatory cytokines in aqueous humor were significantly increased (160–163).

Elevated autoantibody expression is a marker of immune dysregulation in glaucoma, and the cause of autoantibody up-regulation remains unknown. It has been demonstrated that Th17 cells were upregulated and expressed IL-21 in glaucoma patients. These Th17 cells are able to promote Ig secretion by naive B cells in a manner dependent on IL-17A (164).

Microglia, the immunocompetent cells that are activated and respond to neurons after external stimuli (165). Interestingly, microglia changed their morphology after activation and gradually evolve into amoeboid cells that act as macrophages. They migrated to damaged sites, proliferates, engulfs microorganisms, and subsequently damaged tissues (166–169). Excessive activation of microglia released a range of inflammatory cytokines, ultimately leading to damage to neural tissue (90, 170). At the same time, toxic substances from injury triggered the activation of microglia in turn so that inflammatory factors caused apoptosis of RGCs by intrinsic and extrinsic pathways again, which formed a vicious cycle and exacerbated neurodegenerative processes (171, 172). In addition, activated microglia were able to transmit antigens to activated T cells in the retina and optic nerve, thus participating in the process of T cell-mediated neuroprotective immunity and immunopathological damage (173, 174). A significant increase in IL-17A expressed by microglia was found in unilateral laser-induced ocular hypertension mice (31).Using a mouse model of retinal ischemia-reperfusion induced by acute intraocular pressure elevation, the migration and activation of microglia caused up-regulated expression of IL-17A and IL-1β (175). In addition, pressurization of neural cells PC12 mimicked elevated intraocular pressure *in vivo* and IL-17A was found to be promoted in cells after induction (176).

Glaucoma is a blinding disease whose pathogenesis is not yet fully understood, so further studies are needed to understand the role of IL-17A in glaucoma.

## Conclusion

**Figure 3:**
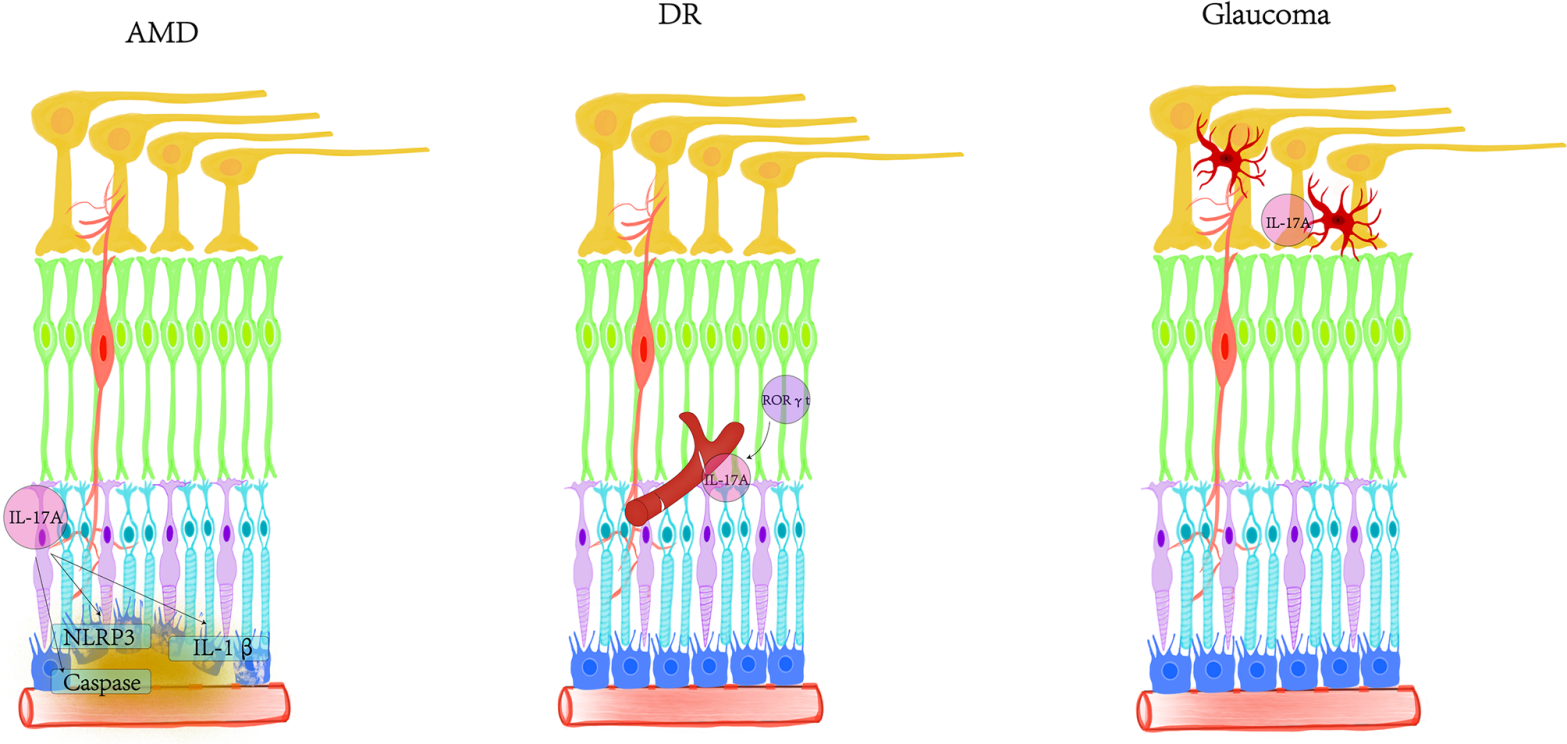
Schematic diagram of the involvement of IL-17A in AMD, DR, and glaucoma. In AMD, IL-17A activates NLRP3, secretes IL-1β, and induces various signaling pathways in RPE. IL-17A promotes the expression of caspase-1, caspase-3 and caspase-9 pathways from the post-transcriptional level. IL-17A disrupts the distribution of tight junctions and destroys the blood-retinal barrier. IL-17A mainly targets blood vessels in DR, inducing inflammation, vascular leakage and capillary degeneration. The cells expressing RORγt may be the main producers of IL-17A in the retina. In glaucoma, IL-17A was found to be closely related to activated microglia.

Currently, IL-17A is the factor most closely related to human health and disease in the IL-17 family. There is a synergistic effect between the development and progression of various retinal degenerative diseases and the imbalance of IL-17A. IL-17A not only participates in inflammatory pathogenicity, but also induces innate immune defense. The functions of IL-17A are more diverse than originally discovered. We still lack understanding of the role of IL-17A in tissue damage. With age, the immune privilege mediated by the retina may gradually weaken and disappear. The reduction in passive protection makes the retina more vulnerable to damage such as oxidative stress in AMD, hyperglycemia in diabetes and ocular hypertension in glaucoma (**Figure 3**). It is necessary to further study how IL-17A interacts with different cells and cytokines in the retina to find better targets and pathways. In the future, intravitreal injection or non-invasive eye drop nanomaterials can be used to encapsulate IL-17 neutralizing antibodies as new treatment methods. Treatment strategies aimed at reducing the production of inflammatory factors may have a beneficial impact on the management of retinal degenerative diseases.

## Author Contributions

HZ wrote the manuscript. XS provided support, supervised, and edited the manuscript. All authors read and approved the final manuscript.

## Funding

This study received by support from the National Natural Science Foundation of China (No. 82171076).

## Conflict of Interest

The authors declare that the research was conducted in the absence of any commercial or financial relationships that could be construed as a potential conflict of interest.

## Publisher’s Note

All claims expressed in this article are solely those of the authors and do not necessarily represent those of their affiliated organizations, or those of the publisher, the editors and the reviewers. Any product that may be evaluated in this article, or claim that may be made by its manufacturer, is not guaranteed or endorsed by the publisher.

## Abbreviations

IL-17A, nterleukin-17A; AMD, Age-related macular degeneration; DR, Diabetic retinopathy; PDR, Proliferative diabetic retinopathy; Aβ, Amyloid-beta peptides; Th, T helper; THP-1, Human myeloid leukemia mononuclear cells; TNF-α, Tumor Necrosis Factor-α; IL-1β, Interleukin-1β; SEFEX, SEFIR-Extension”; CBAD, C/EBP β activation domain; ERK, Extracellular regulated protein kinases; NF-kB, Nuclear factor kB; Bcl-2, B cell lymphoma/lewkmia-2; Sox2, Sex determining region Y-box 2; Oct4, Octamer-binding transcription factor 4; TRAF, TNF-receptor–associated factor; MAPK, Mitogen-activated protein kinase; AP-1, Activating protein-1; MEKK3, Mitogen-activated protein kinase kinase kinase 3; MEK5, Mitogen-activated protein kinase 5; MMP, Matrix metalloproteinases;’ CFH, Complement factor H; C1INH, C1-inhibitor; CFB, Corrugated fiber board boxes; C4bp, C4-binding protein; RPE, Retinal pigment epithelial cells; DEGs, Differentially expressed genes; DMCs, Differentially methylated CpGs; CECs, Choroidal endothelial cells; BRB, Blood-retinal barrier; JAK1, Janus kinase-1; HG, High glucose; RNV, Retinal neovascularization; STZ, Streptozotocin; FADD, Fas-associated protein with death domain; VEGF, Vascular endothelial-derived growth factor; RGCs, Retinal ganglion cells; PD, Parkinson’s disease; ALS, Amyotrophic lateral sclerosis; MS, Multiple sclerosis.
